# A Simulation-Based Comparison of Human, Porcine and Ovine Pulmonary Artery Hemodynamics. Evaluating the Suitability of Large Animal Models for Endopulmonary Device Evaluation from a Hemodynamics Point of View

**DOI:** 10.1007/s13239-025-00803-z

**Published:** 2025-09-02

**Authors:** Pavlo Yevtushenko, Titus Kuehne, Jan Bruening, Leonid Goubergrits

**Affiliations:** 1 Institute of Computer-assisted Cardiovascular Medicine, Deutsches Herzzentrum der Charité (DHZC), Berlin, Germany; 2 Charité – Universitätsmedizin Berlin, corporate member of Freie Universität Berlin and Humboldt-Universität zu Berlin, Berlin, Germany

**Keywords:** Pulmonary artery pressure sensor, Wall shear stress, Oscillatory shear index, Computed tomography, Computational fluid dynamics

## Abstract

In the field of cardiovascular device development, new devices such as heart valves, stents or pressure probes for long term heart failure monitoring are subject to animal trials to evaluate their safety and efficacy. For such applications, swine and sheep are the animal models of choice owed to their similarities to humans with regards to heart size, weight and ventricular kinetics. However, clinical aspects regarding the choice of animal model revolve mainly around anatomical similarities as well as the ability to induce the desired pathology. In the case of pulmonary artery pressure sensors, both swine and sheep appear to be suitable candidates for animal trials since both animals have been used for pre-clinical evaluation. Hemodynamic aspects however, although equally important for device performance, appear rather underrepresented in current research and it remains uncertain whether anatomical similarities between humans and animal model in the region of interest translate to hemodynamic similarities. To provide insight whether pulmonary artery hemodynamics in large animal models are indeed comparable to those in humans, this work presents a computational fluid dynamics-based study on pulmonary artery hemodynamics for humans, swine and sheep. A total of 28 human, 41 porcine and 14 ovine transient simulations of pulmonary artery hemodynamics were performed based on subject-specific geometries reconstructed from computed tomography data. The distributions of wall shear stress (WSS) and oscillatory shear index (OSI) within the cohorts were then compared to assess hemodynamic similarity. Distributions of time averaged WSS were found to be similar between humans and sheep (median 1.2 vs. 1.5 Pa, interquartile range (IQR) 0.8 Pa vs. 0.6 Pa, Wilcoxon rank sum test *p* = 0.42) but were significantly different for swine (median 1.7, IQR 0.5, *p* < 0.05), whereas OSI was significantly different for sheep and swine (0.17 ± 0.04 vs. 0.14 ± 0.03 and 0.09 ± 0.02). between sheep and humans. In summary, pulmonary artery vessel wall stresses of both animal models appear broadly similar to humans, however, sheep seem to have a notable edge over swine in our study.

## Introduction

Large animal models play an important role in cardiovascular research and medical device development and certification. In particular, swine and sheep are the animals of choice due to their similar body and heart weight, heart rate and ventricular kinetics [1–3]. Prominent applications of animal models in the field of cardiovascular disease include myocardial infarction [4, 5], pulmonary hypertension [6], diastolic heart failure [7, 8] as well as the pre-clinical evaluation of medical devices such as ventricular assist devices, endovascular stents and artificial heart valves [1, 9–11]. However, neither sheep nor swine can be considered “perfect” animal models in the sense that findings gained from animal experiments with swine or sheep models are fully applicable to humans. On the one hand, the pathogenesis in animal models is fundamentally different since cardiovascular diseases in animal models do not develop and progress naturally but are induced through diet, medication and/or surgical means, which in turn can lead to unwanted comorbidities or an inability to fully reproduce the desired cardiovascular dysfunction [2, 4, 6, 12]. On the other hand, there are significant differences in cardiovascular anatomy and hemodynamics between swine, sheep and humans [1, 13]. The latter aspect is critical for endovascular device evaluation where cardiovascular anatomy is crucial for device implantation procedures and hemodynamic factors such as wall shear stress (WSS) and oscillatory shear index (OSI) influence device performance and the occurrence of adverse events such as thrombus formation or device migration [14–16]. It is therefore important to ensure that the implantation site of the endovascular device in the animal model is representative in terms of both anatomy and hemodynamics to be able to transfer results obtained from an animal trial to an application in humans. Given the diverse cardiovascular anatomy of both human and animal, the question of sufficient geometric and hemodynamic similarity between animal model and potential patient can only be answered for a specific use case and medical device. Even then, assessing hemodynamic similarity is challenging since hemodynamic parameters, especially those related to the vessel wall, are difficult to quantify in vivo. However, detailed anatomical data combined with computational fluid dynamics (CFD) could provide insight into vascular hemodynamics and thus allow for a quantitative comparison between animal model and human hemodynamics. As an example for such a CFD based similarity assessment, this study evaluates pulmonary artery hemodynamics of swine, sheep and humans with the use case of a pre-clinical evaluation of endopulmonary devices such as a pulmonary artery pressure sensor (PAPS) in mind. PAPS are small endovascular probes which are implanted into the pulmonary artery to support chronic heart failure management through a continuous remote monitoring of pulmonary artery pressure [17]. Currently, there are two FDA certified PAPS devices, the CardioMEMS™ and the Cordella™ HF systems, which used different animals for pre-clinical device evaluation (swine for CardioMEMS, sheep for Cordella) [18, 19], suggesting that there is no consensus on which animal model is best suited for animal trials of PAPS devices.

Naturally, vascular hemodynamics are strongly influenced by the geometry of the vessels themselves. Thus, geometric similarity between human and animal model vessel might suggest hemodynamic similarity and a direct evaluation of hemodynamics would appear obsolete. However, geometric similarity might not be straightforward to define and quantify. Studies on human and large animal pulmonary artery morphometry have been conducted but are mainly focused on perfusion aspects, age related scaling and branching hierarchy [20–22]. These aspects might be insufficient to derive hemodynamic similarity since they omit parameters important from a fluid dynamics point of view such as local radii, tapering and segment lengths. Furthermore, the interaction between shape and hemodynamics are governed by non-linear differential equations, which do not allow to easily assess how observed differences in anatomy translate into functional aspects.

A recent study did perform a more in-depth evaluation of porcine, ovine and human pulmonary artery morphometry and found ovine pulmonary arteries to be more similar to human pulmonary arteries and therefore potentially better suited for animal trials of endopulmonary devices, even though both animal models have been found to feature significant differences with respect to the human anatomy [23]. However, whether the superior geometric similarity of sheep vs. swine translates to a better hemodynamic similarity remains hypothetical since only geometry was considered. For a definitive comparison, other factors governing pulmonary hemodynamics such as heart rate, stroke volume and the resulting local flow velocities as well as derived parameters such as WSS and OSI on the vessel wall need to be considered.

Using the geometry data from the aforementioned morphometry study of sheep, swine and humans, numerical blood flow simulations are performed and relevant hemodynamic parameters are compared. To facilitate a realistic simulation setup and include the influence of species-specific ventricular kinetics, simulations are performed using subject-specific inflow waveforms based on either in-vivo subject-specific measurements (where available) or a species-specific waveform model derived from available clinical or animal data. Based on the simulation data, this study aims to provide a comprehensive evaluation of the suitability of porcine and ovine models for PAPS animal trials from a hemodynamics point of view.

## Results

**Fig. 1:**
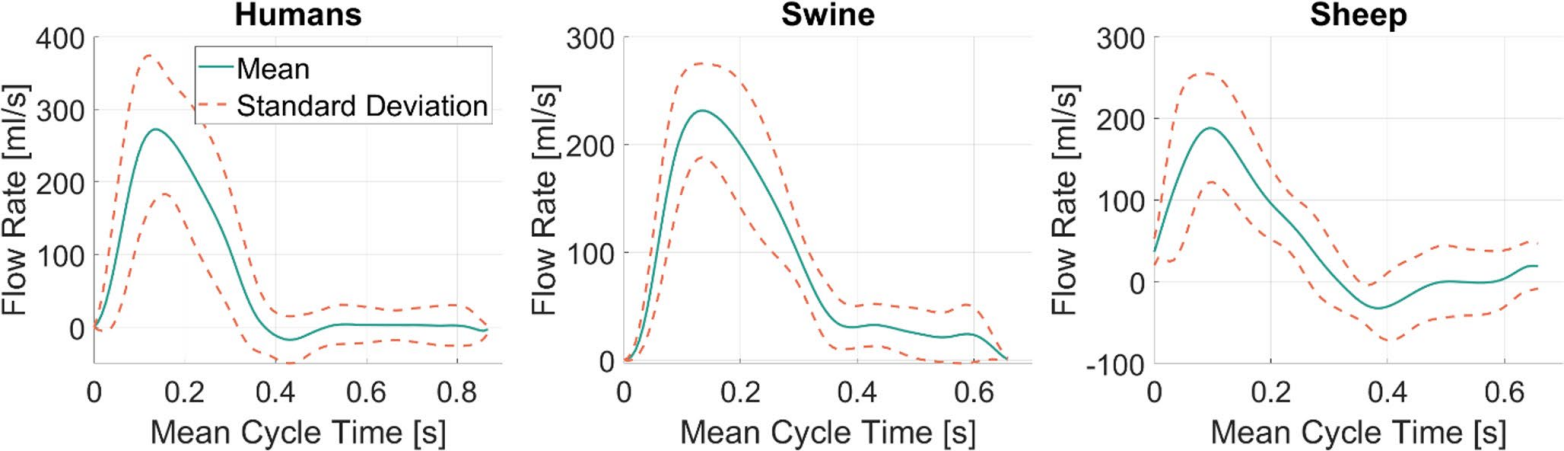
A qualitative comparison of the inlet waveforms of humans, swine and sheep. The mean waveform is plotted in green for each cohort with a dotted red line showing the standard deviation of the individual waveforms from the mean waveform. The mean cycle time was computed as the average cycle time of the respective cohort’s individual waveforms

**Inlet Waveforms.** A qualitative comparison of human, porcine and ovine inlet waveforms is presented in Fig. 1. Here, the mean waveform as well as the standard deviation of waveforms are plotted for the three cohorts. Both animal inlet waveforms show lower peak flows and shorter cycle times. The porcine cohort, however, appears more similar in shape to the human one while also more closely matching peak flow rate (PF) at 230 ml/s vs. 270 ml/s.

**Fig. 2:**
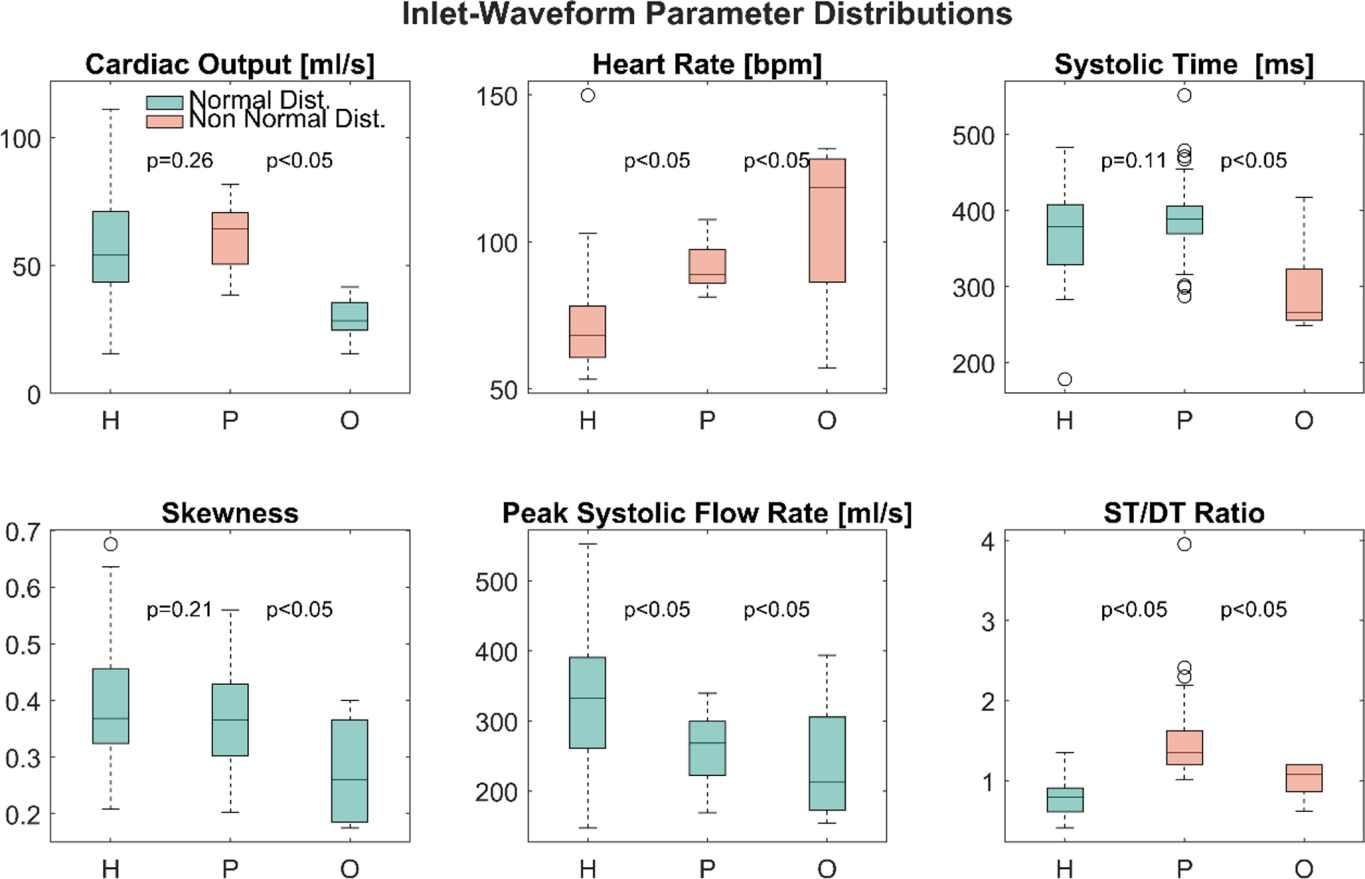
Boxplots of inlet-waveform parameter distributions for the human (H), porcine (P) and ovine (O) cohorts. Green boxes denote a normal distribution of the respective parameter. Outliers, defined as values above/below the 25^th^/75^th^ percentile by more than 1.5 times the inter-quartile range, are marked with an ‘o’ symbol

A quantitative comparison of the inlet waveform parameter distributions is presented in Fig. 2 in the form of boxplots. All parameters were found to be normally distributed except for the heart rate (HR), cardiac output (CO) in the porcine cohort, systolic time (ST) in the ovine cohort and the systolic to diastolic time ratio (ST/DT) in the ovine and porcine cohorts. Significant differences in mean (for normally distributed parameters) and median (for non-normally distributed parameters) values were found for all parameters in the ovine cohort.

For the porcine cohort, the median of CO as well as the means of ST and systolic skewness (SK) did not show statistically significant differences to the human cohort (*p* = 0,26, *p* = 0.11 and *p* = 0.21 respectively). Parameter variances, however, were significantly different for all parameters and both cohorts.

Mean/Median differences were consistently higher for the ovine cohort (*p* < 0.05) as were differences in IQR, except for ST and PF. Here, the ovine cohort showed better agreement with the human one, although median differences remained significant. In general, the porcine cohort’s inflow-waveform parameters showed better agreement with the human cohort’s regarding distribution means/medians. Parameter ranges in contrast appear to be more similar in the ovine cohort. With the exception of CO and SK, the parameters found in the ovine cohort cover 50% or more of the respective human parameter distribution. For swine, only ST and SK covered 50% of the human parameter range. Table 1 summarizes the findings regarding statistically significant differences and range coverage. The raw values for means/medians and standard deviations/IQRs can be found in table 2 . 

**Table 1:** Similarity of inlet-waveform parameter distributions between human and animal cohorts. For parameter means, ‘+’ denotes no significant difference to the human cohort. For parameter ranges, ‘++’ denotes that 75% or more of the human parameter range is covered by the respective animal cohort, ‘+’ denotes a coverage of 50–75% while ‘-‘ indicates a parameter coverage between 25% and 50%. The Raw values for mean/median and ranges can be found in Table 3 in the supplemental section

Parameter	Porcine vs. human			Ovine vs. human	
	Mean	Range		Mean	Range
**Cardiac Output**	+		-	-	-
**Heart Rate**	-		-	-	++
**Systolic Time**	+		+	-	+
**Skewness**	+		++	-	-
**Peak Flow**	-		-	-	+
**ST/DT Ratio**	-		-	-	+
**Legend**					
**- (mean/range)**	Significant difference in mean / coverage between 25% and 50%				
**+ (mean/range)**	No significant difference in mean / coverage between 50% and 75%				
**++ (range only)**	Coverage above 75% of human range				

**Table 2:** Inlet waveform parameter statistics. Mean ± standard deviation is shown for normally distributed parameters. Median and interquartile range (IQR) is shown for non-normally distributed parameters

Parameter	Human Cohort	Porcine Cohort	Ovine Cohort
Cardiac Output [ml/s]	58 ± 20	64, IQR 20	29 ± 7
Heart Rate [bpm]	68, IQR 17	89, IQR 11	118, IQR 42
Systolic Time [ms]	366 ± 63	389 ± 52	266, IQR 68
Systolic Skewness	0.40 ± 0.11	0.36 ± 0.09	0.28 ± 0.08
Peak Systolic Flow Rate	328 ± 96	263 ± 46	234 ± 71
Systolic-to-Diastolic Time Ratio	0.77 ± 0.22	1.35, IQR 0.42	1.08, IQR 0.33

**Fig. 3:**
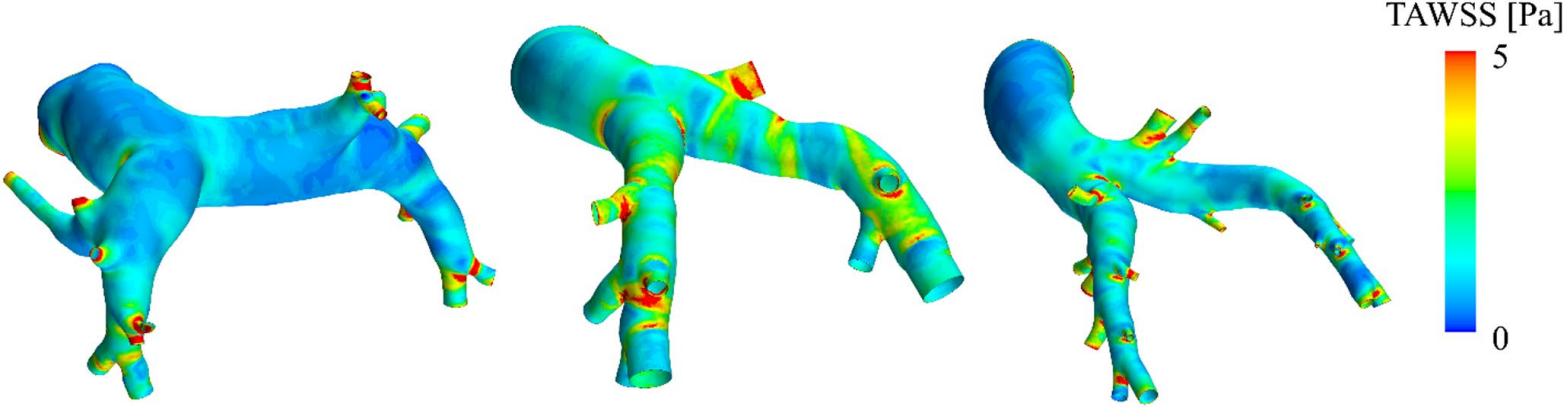
Contours of time averaged wall-shear-stress (TAWSS) for a human (left) a porcine (middle) and an ovine (right) case

**Fig. 4:**
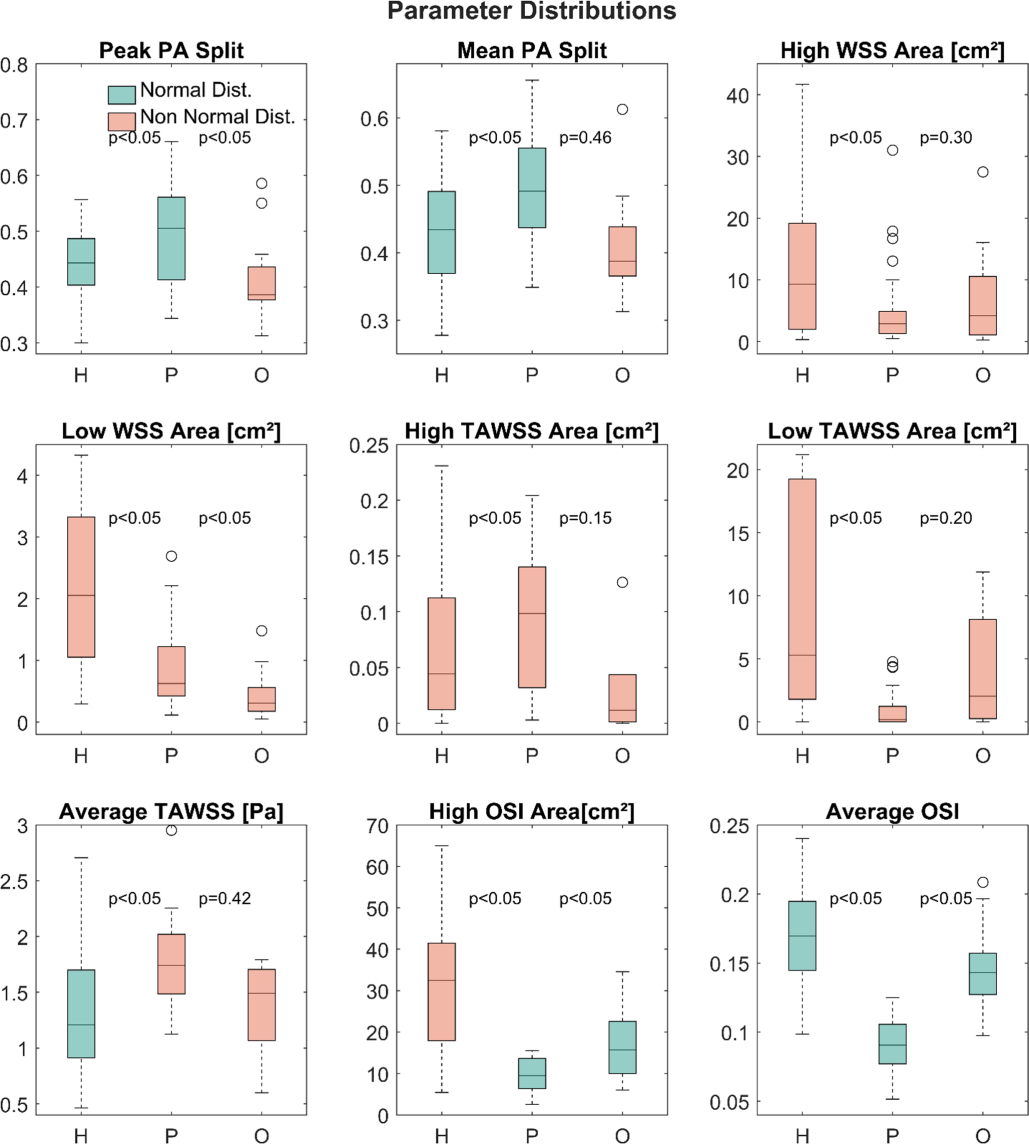
Boxplots of flow splits and shear stress-derived parameter distributions for the human (H), porcine (P) and ovine (O) cohorts: Peak pulmonary artery (PA) flow splits, areas of low and high time averaged and peak wall-shear-stresses (TAWSS/WSS), areas of high oscillatory shear index (OSI) and area averaged TAWSS and OSI. Green boxes denote a normal distribution of the respective parameter. Outliers, defined as values above/below the 25th /75th percentile by more than 1.5 times the inter-quartile range, are marked with an ‘o’ symbol

**Flow splits and shear stress parameters.** Based on the CFD simulations results, time averaged and instantaneous wall stresses are compared between the three cohorts. Figure 3 shows an example of time averaged wall shear stress (TAWSS) distributions on the vessel wall of a human, a porcine and an ovine PA. Figure 4 shows a comparison of the distributions of LPA/RPA flow splits and the shear stress-derived parameters using boxplots colored by sample distribution type (normal/non normal). In contrast to the inflow parameters, most of the parameters evaluated here were not normally distributed. Only average OSI was found to be normally distributed for all cohorts whereas pulmonary artery (PA) flow split, surface-averaged TAWSS and high WSS area were mixed normally/non-normally distributed between the cohorts. The rest of the parameters were found to be non-normally distributed with a notable skewness of high and low WSS/TAWSS areas towards lower values. For high and low WSS areas, the animal cohorts cover either only half the range of the human cohort or even less, as e.g. in the extreme case of low WSS area, where the sheep cohort ranges from 0.1 cm² to 1.5 cm² (excluding outliers) compared to the human cohort’s 0.3 to 4 cm². 

A similarly high discrepancy can be seen for high WSS area in the porcine cohort, where values range from only 0.5 to 10 cm² while the human cohort stretches from 0.3 to 41 cm². Notable differences are also present in the distribution of high and low TAWSS areas. Again, animal cohort ranges are lower than those of the human cohort, except for porcine high TAWSS area, which shows similar a IQR and range (IQR 0.10 vs. 0.11 cm², range 0–0.20 cm² vs. 0–0.23 cm²). The difference in parameter range between human and porcine low TAWSS area stands out particularly with a range of 0 to 21 cm² against 0 to 3 cm². Ovine low TAWSS area appears more similar to human with no significant difference in median (*p* = 0.2) but are still remarkably off regarding IQR and range (IQR 7 vs. 17 cm², range 0–12 cm² vs. 0–21 cm²).

**Table 3:** Similarity grading for flow splits and shear stress parameters. For parameter averages, a ‘+’ indicates no statistically significant differences between means/medians. ‘o‘ indicates significant differences with the median of the animal cohort still within the interquartile range of the human cohort.‘-‘ indicates that the median of the animal cohort is outside of the interquartile range of the human cohort. For parameter ranges, ‘++’ denotes that 75% or more of the human parameter range is covered by the respective animal cohort, ‘+’ denotes a coverage of 50–75%, ‘-‘ indicates a parameter coverage below 50% and ‘--‘ indicating below 25% coverage.

Parameter	Porcine vs. human		Ovine vs. human	
	Average	Range	Average	Range
**Peak PA Split**	-	++	-	+
**Mean PA Split**	-	++	+	+
**High WSS Area**	o	--	+	-
**Low WSS Area**	-	-	-	--
**High TAWSS Area**	o	++	+	--
**Low TAWSS Area**	-	--	o	+
**Average TAWSS**	-	+	+	+
**High OSI Area**	-	--	-	-
**Average OSI**	-	--	o	+
**Legend**				
**-- (range only)**	Coverage below 25% of human range			
**- (average/range)**	Average outside of human IQR / coverage between 25% and 50%			
**o (average only)**	Significant difference in mean or IQR but with mean/median inside human IQR			
**+ (average/range)**	No Significant difference in mean or median / coverage between 50% and 75%			
**++ (range only)**	Coverage above 75% of human range			

**Table 4:** Flow splits and shear stress parameter statistics. Mean ± standard deviation is shown for normally distributed parameters. Median and interquartile range (IQR) is shown for non-normally distributed parameters. Abbreviations: Wall-Shear-Stress (WSS), Time-averaged Wall-Shear-Stress (TAWSS), oscillatory shear index (OSI)

Parameter	Human Cohort	Porcine Cohort	Ovine Cohort
Peak Systolic Flow Split	0.45 ± 0.06	0.49 ± 0.09	0.39, IQR 0.06
Time Averaged Flow Split	0.42 ± 0.11	0.49 ± 0.08	0.39, IQR 0.07
Area of High WSS [cm²]	9.3, IQR 17.2	2.9, IQR 3.6	4.2, IQR 9.4
Area of Low WSS [cm²]	2.1, IQR 2.3	0.63, IQR 0.80	0.31, IQR 0.38
Area of High TAWSS [cm²]	0.04, IQR 0.10	0.10, IQR 0.11	0.01, IQR 0.04
Area of Low TAWSS [cm²]	5.3, IQR 17.5	0.19, IQR 1.21	2.0, IQR 7.9
Surface Averaged TAWSS [Pa]	1.32 ± 0.57	1.74, IQR 0.54	1.49, IQR 0.64
Area of High OSI	32.5, IQR 23.5	9.8 ± 3.7	16.9 ± 8.5
Surface Averaged OSI	0.17 ± 0.04	0.09 ± 0.02	0.14 ± 0.03

Similarly, medians of high WSS area and high TAWSS area for the human and ovine cohorts were not found to be significantly different (*p* = 0.3 and *p* = 0.15), whereas swine has significantly different medians for both parameters. Surface-averaged TAWSS distributions appear mostly similar in both animal cohorts, albeit with significantly different medians between humans and swine (no significant difference for sheep at *p* = 0.42) as well as notably lower ranges for the animal cohorts (0.5–2.7 Pa vs. 1.1–2.3 Pa and 0.6–1.8 Pa). Average OSI distributions are also close between humans and sheep (mean 0.17 ± 0.04 vs. 0.14 ± 0.03, range 0.1–0.24 vs. 0.1–0.2) but show significantly different mean values. Porcine average OSI in contrast shows a significantly lower mean of 0.09 ± 0.02. Distributions of high OSI area are following the previously seen trend with substantially lower ranges and IQRs in the animal cohort (IQR 23 cm² vs. 7 cm² (swine) and 12 cm² (sheep), range 6–65 cm² vs. 3–16 cm² and 6–35 cm²).

The above findings are summarized in the form of a parameter distribution similarity grading in Table 3. Here, the similarity of distribution location (i.e. median/mean) is graded as good (‘+’), moderate (‘o’) or poor (‘-‘) while range coverage is graded as either very good (‘++’, more than 75% coverage), good (‘+’, 50–75% coverage), poor (‘-‘, 25–50% coverage) or very poor (‘--‘, below 25% coverage).The raw values for means/medians and standard deviations/IQR are presented in table 4.

In general, the ovine cohort resembles the human cohort substantially better in terms of most TAWSS-derived parameters and the average OSI. However, the ovine cohort still performs rather poorly when comparing low WSS and high TAWSS area distribution locations and parameter ranges. Mixed results are seen in general for parameter range coverage. The ovine cohort fails to cover more than 75% of human parameter range for any of the parameters evaluated. PA splits, Low TAWSS area and Average TAWSS had a coverage above 50% but below 75% while Low WSS Area and High TAWSS area were poorly covered by the ovine cohort (< 25%). The swine cohort showed good coverage (> 75%) for PA splits and High TAWSS area parameters but only moderate to poor coverage for the rest, with exceptionally poor coverage (< 25%) for the parameters of High WSS area, Low TAWSS area and average OSI.

## Discussion

Since endovascular devices are not only subject to hemodynamic stresses themselves but are also altering near wall hemodynamics, vascular hemodynamics play a key role in the performance and safety of these devices. Adverse events in the form of thrombus formation and vessel wall damage are therefore not only influenced by the anatomy of the vessel and the shape of the device but also by the hemodynamic stresses acting on them. In this context, areas of high and low TAWSS are known to facilitate platelet aggregation and thrombus formation [14, 24, 25], whereas areas of low TAWSS and/or high OSI are associated with atherosclerosis [26, 27]. Given the lack of reliable hemodynamic data on either humans or large animals however, a similarity of hemodynamics and hemodynamically induced stresses on the vessel walls in animal models are only implicitly, if at all, provided through a similarity of the vessel anatomy and cardiac output. To quantify intravascular hemodynamics and assess hemodynamic similarity between species, this study proposes CFD simulations based on high resolution imaging data of the pulmonary artery acquired through computed tomography and/or magnetic resonance imaging. As an example of such a CFD-based evaluation of hemodynamic similarity, this study compared simulations of human, porcine and ovine pulmonary artery hemodynamics to assess the feasibility of large animal trials for endopulmonary device development and evaluation.

The comparison of the hemodynamic boundary conditions in the form of inlet-waveforms at the beginning yielded expectable results. With the swine’s heart being closer in size and cardiac output to the adult human heart compared to sheep, the porcine cohort showed a better agreement compared to the ovine cohort for most of the inlet-waveform parameters evaluated. Nevertheless, significant differences were found between mean heart rates and peak systolic flow rates for the porcine vs. the human cohort. Moreover, parametwer ranges varied substantially between humans and both animal cohorts.

The comparison of the shear stress-derived parameters presented more interesting results in contrast. Here, the ovine cohort appeared more similar regarding most parameters and agreed particularly well with the human cohort for high WSS area, high TAWSS area as well as the surface-averaged TAWSS. For these parameters, no significant differences regarding parameter averages were found. Furthermore, the parameter ranges of the ovine cohort were closer to the human cohort than those of the porcine cohort for all parameters except for two (area of low WSS and area of high TAWSS).

For the evaluation of the synthetic inlet waveforms, four parameters which were not directly prescribed through clinical data but are a result of the variability of the flow curves (i.e. systolic time, skewness, peak flow rates and the ST/DT ratio) are of particular interest. With the sheep waveforms being taken from in-vivo measurements, the comparison between sheep and swine/human can be used to compare real and synthetic inlet waveforms. Here, the variance was similar between humans and sheep, whereas swine showed notably narrower distributions of systolic time and peak flow rates. This may be the result of the overall lower cardiac outputs and heart rates found in the swine cohort or indicate a low variance in the training data for the swine waveform SSM or, most likely, a combination of both.

Overall, ovine pulmonary arteries appeared to be a better choice for pre-clinical animal trials of endopulmonary devices with respect to hemodynamically induced vessel stress. This may appear surprising at first given the result of the inlet-waveform comparison. However, a morphometric analysis previously performed on the very cohorts used for the simulations performed here found that the ovine cohort features a stronger geometrical similarity to the human cohort. In particular, no significant differences in RPA/LPA lengths, as well as curvature and ellipticity indices were found between human and sheep, whereas swine showed only a good agreement for RPA length [23]. Therefore, the superior inlet-waveform similarity of the porcine cohort is apparently not able to compensate for an inferior geometrical similarity. It should be noted however that swine inlet flow waveforms showed a notably lower variability than human and sheep waveforms in our data, which may have skewed the results in favor of the ovine cohort regarding parameter ranges.

For the pre-clinical evaluation of endopulmonary devices, it is critical to ensure that the near wall flow field is not altered by the endopulmonary device in such a way that promotes endothelial damage and/or thrombus formation. In this context, the animal model should resemble human near-wall hemodynamics as closely as possible to accurately predict any possible adverse effects the device may have on the vessel wall or near wall flow. To that end, the ovine model appears to be more suitable than the porcine model. However, device size and implantability may necessitate the use of swine, in which case one must be aware of potential discrepancies between human and porcine PA hemodynamics, wall stresses and shapes. Most notably, the area of low TAWSS, a risk factor for thrombus formation, appears highly underestimated in the porcine animal model.

Regarding sample sizes, it is worth noting that the ovine cohort, despite having half the size of the human cohort, yielded parameter ranges similar to human ones for all but two shear stress parameters. This suggests that even small cohorts at the order of ten might provide sufficient variability regarding the magnitude and distribution of hemodynamic stresses. This is further supported by recent FDA certification studies for endopulmonary pressure sensors which used 9 pigs and 28 sheep respectively [18, 19]. Considering the high costs and ethical objections associated with studies involving large animals, reducing cohort sizes is imperative and the results obtained here might provide some guidance for future studies on how many animals are necessary to obtain meaningful data. Beyond evaluating hemodynamic parameters and shear stresses of interest, CFD simulations can also be utilized to explore the feasibility of implanting multiple devices in one animal to further reduce cohort size by predicting potential interaction effects.

Beyond the specific results obtained from the simulations, this study presents a use-case example for CFD frameworks in medical device design and certification. Modelling and simulation approaches are increasingly used and encouraged by regulating bodies such as the FDA [28]. In the case of endopulmonary device evaluation, the methods presented here could aid cohort size estimation and animal model choice by giving an a-priori estimation on the distributions of the hemodynamic parameters of interest in both humans and animal models. Moreover, the expected response of PA hemodynamics to an alteration of ventricular dynamics in the animal model through pacing or medication can be estimated, further aiding the animal study design.

Finally, it should be noted that the results regarding the areas of high and low WSS/OSI are highly dependent on the threshold chosen for high and low values. Currently, there appears to be no agreement on distinct WSS ranges that do not promote endothelial damage and/or thrombosis [29–32]. However, TAWSS values between 1.5 and 2.5 Pa are generally agreed upon to be athero- as well as thromboprotective and previous studies on vessel thrombosis and endothelial damage suggest 0.4 Pa as a lower bound for risk-free TAWSS [29, 30], which was also used in this study. On the other end, TAWSS beyond 10 Pa are associated with endothelial damage and thrombosis [33] and a conservative threshold of 15 Pa was used in this study. These literature based best estimates may not be suitable for a different use case, for which other thresholds may tip the favor towards swine or further towards sheep. However, given the fact that the distributions of average TAWSS and OSI agreed better for sheep, the ovine cohort is likely to perform equally well for different thresholds compared to the porcine cohort.

### Limitations

While the above findings appear conclusive, some limitations of the study design need to be considered. The first are necessary simplifications to the physics modelling in the form of rigid walls and a constant pressure boundary condition at the outlets of the left and right pulmonary arteries. Rigid wall simulations are known to overestimate WSS [34–36]. However, rigid-wall and fluid-structure-interaction based simulations show strong correlations for WSS and given the comparative nature of this study, the rigid-wall assumption does not incriminate its main findings. Moreover, we do not expect an increased uncertainty in our results due to inter-species differences in vessel distensibility as previous studies have found both porcine and ovine PA distensibility to be similar to humans [37, 38].

Using constant pressure outlet boundary conditions instead of a more sophisticated Windkessel model to account for downstream compliance may be seen as potential shortcoming. However, it is the most reasonable choice for this study, even when setting aside the reduced computational costs. The shear-stress parameters evaluated here are all derived from the velocity field. The velocity field itself is mainly governed by the inlet waveform and the LPA/RPA flow splits, the latter being a result of pressure differences between the individual outlet boundaries. The absolute values of pressure do not influence the solution of the rigid-wall, incompressible flow field. Assuming that there are no obstructions or malformations in the vascular structure between the simulated domain and the capillary bed, the pressure downstream of the simulated domain will be roughly equal in all branches at any given time point. The pressure boundary condition used here adheres to that by setting the same pressure for all outlet boundaries throughout the cardiac cycle, resulting in physiologically sound LPA/RPA flow splits and therefore shear-stresses. This is further supported by additional simulations carried out with three-element Windkessel boundary conditions using model parameters für healthy PA published by Zambrano et al. [39]. Here, notable differences were found for pressure values at the in and outlets. However, no substantial changes could be observed in the flow splits or the velocity field as well as any of the parameters investigated (i.e. WSS and OSI). This does not mean that the constant pressure boundary condition used here can be seen as generally suitable for any PA simulations. However, within the context of this study, which focuses on flow and velocity-derived shear stresses, it is sufficiently accurate and, considering the absence of any data to parametrize Windkessel boundary conditions, perhaps the only sensible one.

In contrast to outlet boundary pressure, the flow rates prescribed at the MPA inlet have a large impact on the wall stresses evaluated here. Considering this, the allometric model used to calculate swine CO and HR from body weight may be problematic since this approach might have contributed to the relatively small ranges observed for porcine CO and HR. However, while clinically measured values of HR and CO for the porcine cohort might feature stronger variability and would thus increase parameter ranges, parameter means and medians are unlikely to change substantially, leaving sheep superior to swine in that regard. Besides the flow rates themselves, the shape of the inlet velocity profile on the inlet boundary may have an influence on the wall stresses in the MPA. For CFD simulations, there are two common profile types: parabolic and plug. The former one prescribes a parabolic distribution with high velocities in the center of the vessel and zero velocity at the vessel wall, mimicking a fully developed flow in a pipe. The latter prescribes the same velocity on the entire inlet surface. However, neither profiles can be considered accurate for MPA flow, where the pulmonary valve shapes the flow profile. Preliminary studies with a few human cases where in-vivo profile measurements could be obtained suggested that the influence of the inlet profile shape on global wall stress parameters is rather low. However, if the hemodynamics in the proximal part of the MPA are of primary interest, profile shape could play an important role and more accurate inlet boundary conditions might become necessary.

Another aspect is the low sample size of the ovine cohort with 14 subjects compared to 28 and 41 for humans and swine respectively. A larger sample size may level out the differences and diminish the advantage the ovine cohort has with the current data. What can be concluded with a certain confidence however is that geometric similarity appears to be the driving factor for hemodynamic similarity with inlet boundary conditions playing a secondary role. Since it appears rather unlikely that for a larger ovine cohort, geometric similarity would increase while hemodynamic similarity would decrease, the above statement on the primary importance of geometric similarity for hemodynamic similarity holds true for the sample size available here and may be generalized to larger populations. It is also worth noting that due to the high costs involved in large animal studies, the sample sizes available for this study can be considered representative of animal trials of endovascular devices. For instance, the animal trials involved in the FDA approval of the CardioMEMS and Cordella PAPS sensors included 9 pigs and 28 sheep respectively [18, 19].

## Materials and Methods

**Vessel reconstruction.** This study includes hemodynamic simulations of 28 human, 41 porcine, and 14 ovine pulmonary arteries (PA). Porcine PA geometries were reconstructed using electrocardiography-gated computed tomography (CT) scans retrospectively obtained from the ETH Zürich (Swiss Large White, weight 82.6 ± 18.8 kg). These animal studies were approved by the local Committee for Experimental Animal Research (Cantonal Veterinary Office Zurich, Switzerland) under the approval numbers 152/2013, 219/2016 and 138/2017. Image data for the reconstruction of Ovine PA geometries (subject weight 49.1 + 6.9 kg) was obtained from a study at the Research Institute for Experimental Medicine of the Charité. This study was approved by the State Office of Health and Social Affairs Berlin and conducted according to the Federation of Laboratory Animal Science Association’s guidelines. A detailed description of the acquisition procedures can be found in the respective studies [40–42]. For human PAs, image data was obtained from routine transcatheter aortic-valve replacement (TAVI) planning. CT scans were performed with either an Aquilion One Vision (Canon Medical Systems, Tochigi, Japan) or a Revolution CT (GE Healthcare, Chicago, IL, USA) yielding an in-plane resolution of 0.39–0.648 mm x 0.39–0.648 mm and a slice thickness of 0.5–1 mm. Average patient age was 81 ± 7.7 years (60% female). Due to the retrospective nature of the study, the ethical board of the Charité Universtitätsmedizin waived the need of obtaining informed consent. All methods were carried out in accordance with relevant guidelines and regulations. All experimental protocols were approved by the ethical board of the Charité Universtitätsmedizin (approval number EA2/004/21).

**Fig. 5:**
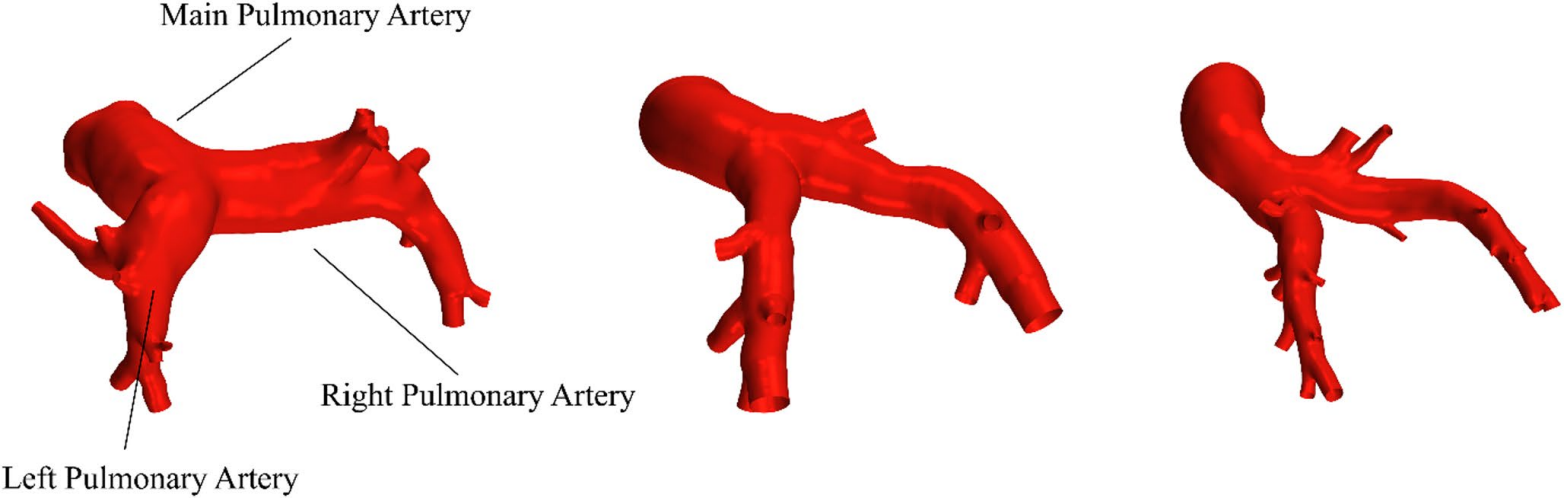
An exemplary set of reconstructed human (left), porcine (middle) and ovine (right) pulmonary artery geometries used to perform hemodynamic simulations

Geometry reconstruction was performed using ZIBAmira (version 2015.28, Zuse Institute Berlin, Berlin, Germany) from images acquired during the diastole using semi-automatic segmentation approach. Briefly, the high contrast areas within the PA were threshold-filled to construct an initial label field in the image stack. This label field was then manually corrected by adding dark areas within the PA that were not filled by the thresholding or remove surplus tissue from the label field which did not belong to the PA. An in-house volume-preserving surface generation tool created the triangulated vessel surfaces from the final, corrected label fields. The resulting PA surfaces were trimmed proximally at the sinus of the pulmonary trunk. At the left and right pulmonary arteries (LPA and RPA), the branching vessels were included in the surface and trimmed distally before the onset of bifurcation. A detailed description of the reconstruction procedure as well as an inter-operator bias analysis for the segmentation of the CT data can be found in an earlier morphometry study [23]. Exemplary geometries of human, porcine and ovine PA are shown in Fig. 5.

**Inflow waveform.** To facilitate a realistic simulation setup, time dependent subject and species-specific flow rates were prescribed at the inlet of the flow domain. However, PA flow measurements were not available for any of the human or porcine subjects. Therefore, a hybrid approach utilizing a principle component analysis of human and porcine PA inflow waveforms, subject-specific heart-rates (HR) as well as cardiac outputs (CO) were used to provide synthetic waveforms where measurements were unavailable. The human waveform models were created using time resolved PA flow rates obtained through 4D magnetic resonance imaging (4DMRI) of a comparable adult human cohort in terms of age and heart rate and cardiac output (*n* = 64, age 67 ± 11 vs. 81 ± 7.7, HR 65 ± 9 vs. 72 ± 18 bpm 76 ± 19 vs. 58 ± 20 ml/s) acquired in an earlier study [43]. While not an exact match, inflow waveforms based on this data provide a more realistic setup than analytically generated waveforms. The porcine waveform SSM was likewise created from data acquired earlier, which included 4DMRI scans of 25 adult swine [44, 45].

**Fig. 6:**
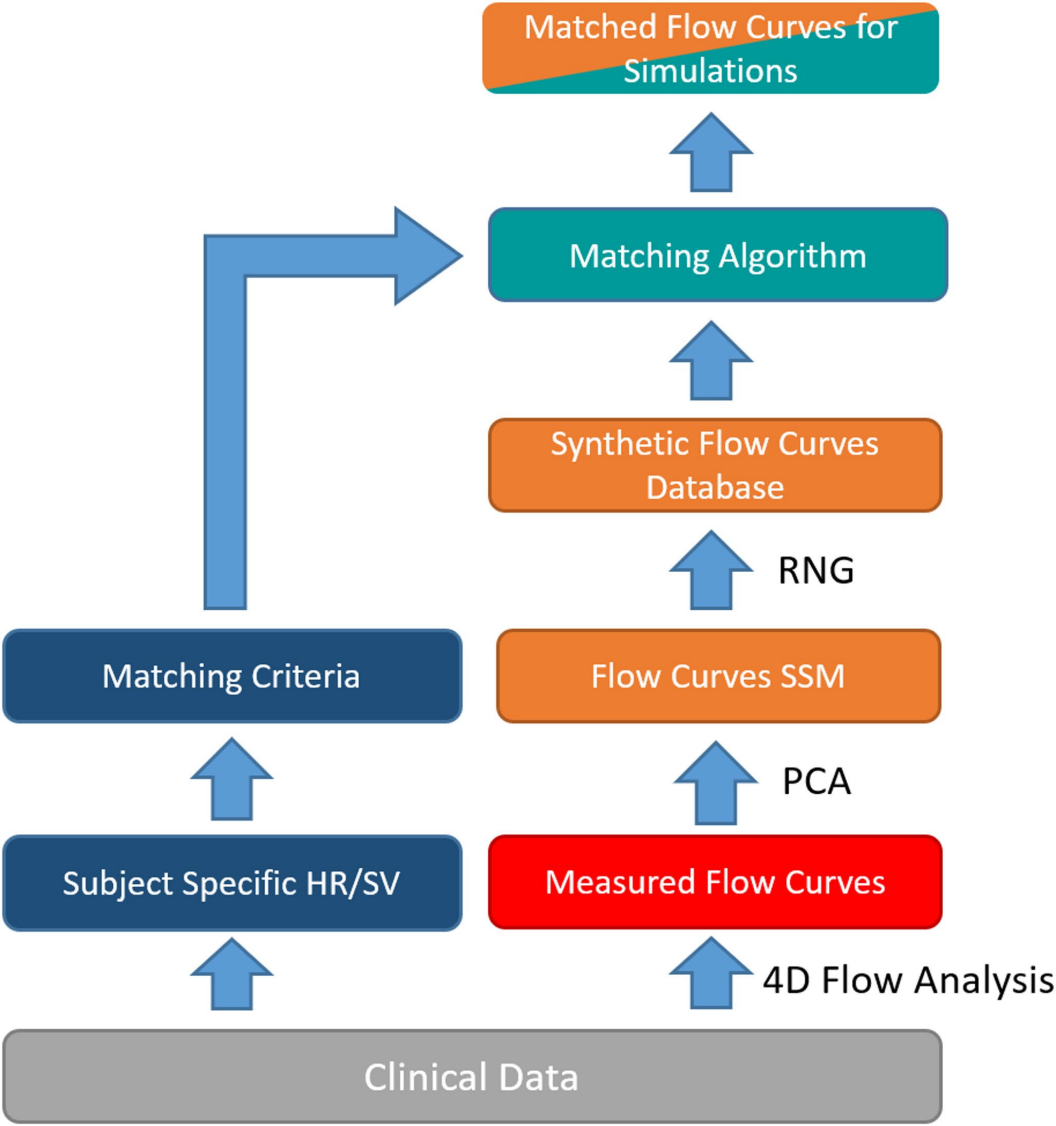
Synthetic inlet waveform generation. Patient specific flow rates are measured from 4D magnetic resonance imaging. A principal component analysis (PCA) captures the characteristic variation within the patient-specific waveforms and creates the statistical shape model (SSM) representation, from which a large database of synthetic waveforms is created using a random number generator (RNG). From this database, a subject specific waveform is selected based on a requirement for heart rate (HR) and stroke Volume (SV), which is in turn derived from clinical data for that specific subject

The waveform models were created using a principal component analysis (PCA) on uniformly sampled flow curves with 100 points. Information on heart rate was included by adding an additional data point at the end of each waveform time series containing the cycle length of the individual waveform. By including the cycle length in the waveform data vector, the SSM can capture any relationships between waveform shape and cycle length. The first ten PCA modes were found to be sufficient to replicate the original curves with a mean error below 1% and thus only the first ten PCA modes were used for the synthetic waveforms. After the PCA, each flow curve can be expressed as:

$$\mathop F\limits^ \rightharpoonup = {\mathop F\limits^ \rightharpoonup _{mean}} + EV \cdot \mathop b\limits^ \rightharpoonup$$

where $\mathop F\limits^ \rightharpoonup$ is a given flow curve, ${\mathop F\limits^ \rightharpoonup _{mean}}$ is the mean flow curve, EV the matrix of PCA modes from the Eigenvalue decomposition and $\mathop b\limits^ \rightharpoonup$ the mode weight vector for $\mathop F\limits^ \rightharpoonup$. Using the above representation, a large database of synthetic waveforms was created using randomly sampled weights $\mathop b\limits^ \rightharpoonup$. From this database, flow curves were selected for each subject based on a subject-specific requirement for HR and CO. To avoid duplicate waveforms, any waveform already matched to a subject was excluded from further queries of the synthetic database. While HR and CO were available for the human cohort, these parameters were unknown for the porcine cohort and were therefore calculated based on subject weight using an empirical model that relates body weight to HR and CO [46]. Figure 6 summarizes the process of creating the synthetic inlet waveforms.

**Numerical methods.** Hemodynamic simulations were performed using StarCCM + 18.06 (Siemens PLM, Plano, Texas, USA), a finite volume-based solver using the incompressible Navier-Stokes equations to calculate pressure and velocity fields. Given the relatively high Reynolds numbers anticipated during peak-systole (Re > 5000), the k-ω SST turbulence model was used to account for turbulent effects. Pressure correction was performed using the SIMPLE algorithm and time discretization utilized a second order Euler scheme.

**Fig. 7:**
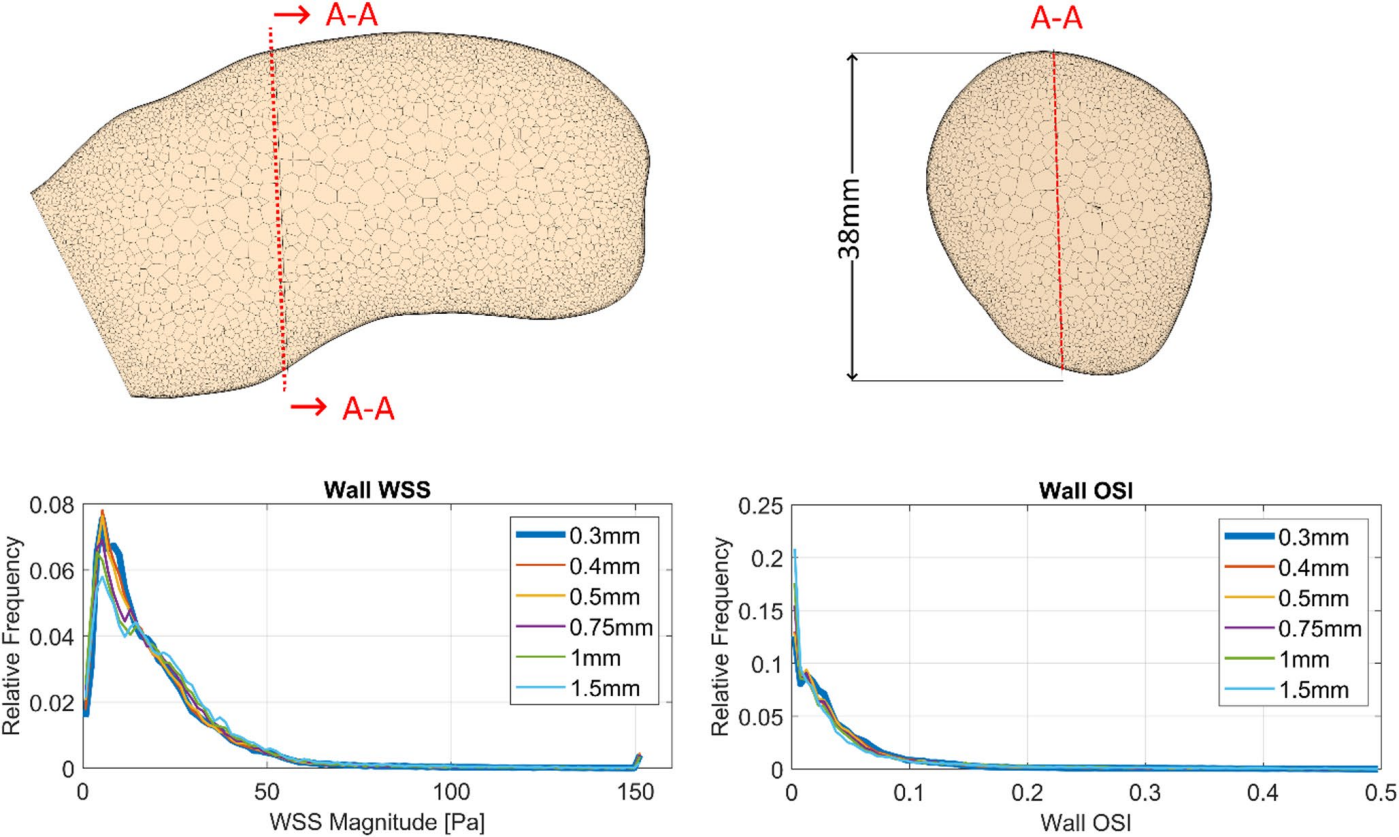
Top: Longitudinal and cross-sectional cuts through a human main pulmonary artery showcasing the computational mesh. Bottom: Results of the mesh sensitivity study depicting time averaged wall shear stress (WSS) and oscillatory shear index (OSI) histograms for the six mesh levels evaluated. The six base sizes of 0.3, 0.4, 0.5, 0.75, 1 and 1.5 mm resulted in mesh sizes of 17, 6.5, 4.2, 1.9, 1.1 and 0.8 million cells respectively

Computational mesh and time-step sizes were determined based on a previous mesh and time-step convergence study. A target cell size of 0.75 mm and a time step of 1 ms were found to yield the best balance between accuracy and computational demand. StarCCM+´s built in polyhedral meshing algorithm was used to create the volume and surface meshes. To improve the velocity resolution near the vessel walls, a six-layer boundary prism layer was created. Figure 7 showcases the mesh structure and resolution in a longitudinal and a cross-sectional cut through a human main pulmonary artery (MPA). Additionally, TAWSS and OSI distributions for six different mesh levels evaluated for the mesh sensitivity analysis are shown.

4DMRI measured (sheep) or subject-matched synthetic inflow waveforms (human and swine) were prescribed at the inlet boundary. For the outlet and wall boundaries, some simplifications were necessary due to a lack of suitable material and lumped element models for swine and sheep on the one hand and to maintain reasonable computational costs for the simulations on the other. At the outlets of the flow domain, a constant pressure of 10 mmHg was set and vessel walls were assumed to be rigid.

**Fig. 8:**
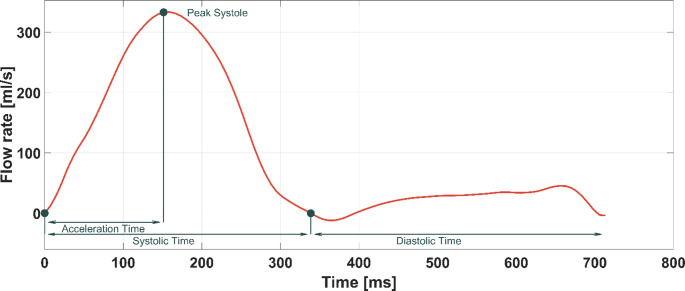
Inflow waveform measuring points. The acceleration time is defined as the time between the beginning of the cardiac cycle (pulmonary valve opening) and the time point at which peak systolic flow is achieved. The systolic time is defined as the time between cycle start and the closing of the pulmonary valve. The systolic skewness is computed as the ratio between these two parameters

**Post Processing.** Both inlet waveforms as well as the resulting PA hemodynamics are compared between the three cohorts. For the inlet waveforms, six parameters are evaluated: Systolic time (ST), systolic skewness (SK), peak systolic flow (PF), heart rate (HR), cardiac output (CO) and the ratio between systolic and diastolic time (ST/DT). Systolic time is defined as the ratio between the acceleration time, which is the time between cycle start and peak systolic flow, and the total cycle time. The diastolic time is the total cycle time minus the systolic time. An example of these measurements on a human PA inflow wave is depicted in Fig. 8.

PA hemodynamics are characterized by a set of scalar values derived from the numerical solution of the velocity and pressure fields. The following parameters are evaluated:

- Peak systole flow rate ratio between left and right pulmonary arteries (LPA and RPA), expressed as the fraction of flow through the LPA.
- Time averaged flow rate ratio between LPA and RPA, expressed as the fraction of flow through the LPA.
- Wall shear stress (WSS) at peak systole.
- Time averaged wall shear stress (TAWSS).
- Oscillatory shear index (OSI).

TAWSS is defined as:

$$TAWSS = \frac{{\sum\limits_1^N {\left| {W\mathord{\buildrel{\lower3pt\hbox{$\scriptscriptstyle\rightharpoonup$}} \over S} {S_i}} \right|} }}{N}$$

where $${\left| {W\mathord{\buildrel{\lower3pt\hbox{$\scriptscriptstyle\rightharpoonup$}} \over S} {S_i}} \right|}$$ is the magnitude of WSS vector at time point *i* and N is the number of time points in a cardiac cycle.

The OSI is defined as:

$$\:OSI=0.5\cdot\:\left(1-\frac{\sqrt{{\left(\frac{\sum\:_{1}^{N}WS{{S}_{x}}_{i}}{N}\right)}^{2}+{\left(\frac{\sum\:_{1}^{N}WS{{S}_{y}}_{i}}{N}\right)}^{2}+{\left(\frac{\sum\:_{1}^{N}WS{{S}_{z}}_{i}}{N}\right)}^{2}}}{TAWSS}\right)$$

where $\:WS{{S}_{x}}_{i}$, $\:WS{{S}_{y}}_{i}$ and $\:WS{{S}_{z}}_{i}$ are the x, y and z-components of the WSS vectors. OSI is a measure of directional change of near wall flow. An OSI of zero implies no change in flow direction at the vessel wall throughout the entire cardiac cycle whereas an OSI of 0.5 results from a periodic reversal of the flow direction near the vessel wall.

Using the values obtained for peak WSS, TAWSS and OSI, additional parameters are defined denoting the amount of vessel area subjected to critical shear stresses, as outlined in the discussion. These parameters are:

- Vessel area with peak WSS above 15 Pa, denoted as high WSS area.
- Vessel area with peak WSS below 0.4 Pa, denoted as low WSS area.
- Vessel area with TAWSS above 15 Pa, denoted as high TAWSS area.
- Vessel area with TAWSS below 0.4 Pa, denoted as low TAWSS area.
- Vessel area with OSI above 0.3, denoted as high OSI area.

Statistical tests were performed using MATLABR 2024b (The MathWorks, Inc., Natick, MA, USA). Sample normality was tested using the Shapiro-Wilk test. If population samples were found to be normally distributed, significant differences in mean and variance were tested using the Student’s t-test and F-test respectively. If samples were not normally distributed, the Wilcoxon rank sum test was used to test median differences. A *p*-value of 0.05 was used for all hypothesis tests.

## Data Availability

All individual surface geometries of the human, porcine, ovine pulmonary arteries as well as the morphometric parameters are made available together with this study: https://figshare.com/articles/dataset/Data_for_CT-based_comparison_of_porcine_ovine_and_human_pulmonary_arterial_morphometry_/21919428. Further datasets used and/or analyzed during the current study are available from the corresponding author upon reasonable request.

## References

[1] D. K. Meyerholz, E. R. Burrough, N. Kirchhof, D. J. Anderson, and K. L. Helke, “Swine models in translational research and medicine,” (in eng), *Vet Pathol*, vol. 61, no. 4, pp. 512–523, Jul 2024, 10.1177/03009858231222235 10.1177/03009858231222235 38197394

[2] N. Milani-Nejad and P. M. Janssen, “Small and large animal models in cardiac contraction research: advantages and disadvantages,” (in eng), *Pharmacol Ther*, vol. 141, no. 3, pp. 235 – 49, Mar 2014, 10.1016/j.pharmthera.2013.10.007 10.1016/j.pharmthera.2013.10.007 PMC3947198 24140081

[3] Y. Suzuki, A. C. Yeung, and F. Ikeno, “The representative porcine model for human cardiovascular disease,” (in eng), *J Biomed Biotechnol*, vol. 2011, p. 195483, 2011, 10.1155/2011/195483 10.1155/2011/195483 PMC3022214 21253493

[4] J. H. Geens, S. Trenson, F. R. Rega, E. K. Verbeken, and B. P. Meyns, “Ovine models for chronic heart failure,” (in eng), *Int J Artif Organs*, vol. 32, no. 8, pp. 496–506, Aug 2009, 10.1177/039139880903200804 19844891 10.1177/039139880903200804

[5] A. Spannbauer et al., “Large Animal Models of Heart Failure With Reduced Ejection Fraction (HFrEF),” (in eng), *Front Cardiovasc Med*, vol. 6, p. 117, 2019, 10.3389/fcvm.2019.00117 31475161 10.3389/fcvm.2019.00117 PMC6702665

[6] J. P. Dignam, T. E. Scott, B. K. Kemp-Harper, and A. J. Hobbs, “Animal models of pulmonary hypertension: Getting to the heart of the problem,” (in eng), *Br J Pharmacol*, vol. 179, no. 5, pp. 811–837, Mar 2022, 10.1111/bph.15444 33724447 10.1111/bph.15444

[7] S. Dubi and Y. Arbel, “Large animal models for diastolic dysfunction and diastolic heart failure-a review of the literature,” (in eng), *Cardiovasc Pathol*, vol. 19, no. 3, pp. 147 – 52, May-Jun 2010, 10.1016/j.carpath.2008.12.008 10.1016/j.carpath.2008.12.008 19211273

[8] C. Miyagi, T. Miyamoto, T. Kuroda, J. H. Karimov, R. C. Starling, and K. Fukamachi, “Large animal models of heart failure with preserved ejection fraction,” (in eng), *Heart Fail Rev*, vol. 27, no. 2, pp. 595–608, Mar 2022, 10.1007/s10741-021-10184-9 34751846 10.1007/s10741-021-10184-9

[9] G. Monreal, L. C. Sherwood, M. A. Sobieski, G. A. Giridharan, M. S. Slaughter, and S. C. Koenig, “Large animal models for left ventricular assist device research and development,” (in eng), *Asaio j*, vol. 60, no. 1, pp. 2–8, Jan-Feb 2014, 10.1097/mat.0000000000000005 10.1097/MAT.0000000000000005 24270232

[10] M. Weisskopf et al., “Dos and don’ts in large animal models of aortic insufficiency,” (in eng), *Front Vet Sci*, vol. 9, p. 949410, 2022, 10.3389/fvets.2022.949410 10.3389/fvets.2022.949410 PMC9478759 36118338

[11] J. P. Carney et al., “New Model for the Assessment of Transcatheter Aortic Valve Replacement Devices in Sheep,” (in eng), *J Invest Surg*, vol. 35, no. 2, pp. 371–377, Feb 2022, 10.1080/08941939.2020.1864796 33371759 10.1080/08941939.2020.1864796

[12] P. Camacho, H. Fan, Z. Liu, and J. Q. He, “Large Mammalian Animal Models of Heart Disease,” (in eng), *J Cardiovasc Dev Dis*, vol. 3, no. 4, Oct 5 2016, 10.3390/jcdd3040030 10.3390/jcdd3040030 PMC5715721 29367573

[13] G. Georges, T. Couture, and P. Voisine, “Assessment of Large Animal Vascular Dimensions for Intra-Aortic Device Research and Development: A Systematic Review,” (in eng), *Innovations (Phila)*, vol. 18, no. 2, pp. 144–151, Mar-Apr 2023, 10.1177/15569845231164134 10.1177/15569845231164134 PMC10159216 37029653

[14] A. K. W. Buck et al., “Combined In Silico and In Vitro Approach Predicts Low Wall Shear Stress Regions in a Hemofilter that Correlate with Thrombus Formation In Vivo,” (in eng), *Asaio j*, vol. 64, no. 2, pp. 211–217, Mar/Apr 2018, 10.1097/mat.0000000000000649 10.1097/MAT.0000000000000649 PMC5823711 28857774

[15] I. Hosaka, T. Uzuka, R. Umeta, and A. Sasaki, “Stent-induced new entry and device migration associated with hemodynamic stress after thoracic endovascular aortic repair for type B chronic aortic dissection using computational fluid dynamics analysis: a case report,” (in eng), *Gen Thorac Cardiovasc Surg Cases*, vol. 3, no. 1, p. 8, Feb 22 2024, 10.1186/s44215-024-00146-6 39516992 10.1186/s44215-024-00146-6 PMC11533579

[16] F. Sturla et al., “Fast Approximate Quantification of Endovascular Stent Graft Displacement Forces in the Bovine Aortic Arch Variant,” (in eng), *J Endovasc Ther*, vol. 30, no. 5, pp. 756–768, Oct 2023, 10.1177/15266028221095403 35588222 10.1177/15266028221095403 PMC10503258

[17] S. P. Radhoe and J. J. Brugts, “CardioMEMS™: a tool for remote hemodynamic monitoring of chronic heart failure patients,” (in eng), *Future Cardiol*, vol. 18, no. 3, pp. 173–183, Mar 2022, 10.2217/fca-2021-0076 10.2217/fca-2021-0076 34697954

[18] (2014). *CardioMEMS HF System SSED*. [Online] Available: https://www.accessdata.fda.gov/cdrh_docs/pdf10/P100045B.pdf

[19] (2024). *Cordella Pulmonary Artery Sensor System SSED*. [Online] Available: https://www.accessdata.fda.gov/cdrh_docs/pdf23/P230040B.pdf

[20] M. Dong et al., “Image-based scaling laws for somatic growth and pulmonary artery morphometry from infancy to adulthood,” (in eng), *Am J Physiol Heart Circ Physiol*, vol. 319, no. 2, pp. H432-h442, Aug 1 2020, 10.1152/ajpheart.00123.2020 10.1152/ajpheart.00123.2020 PMC7473930 32618514

[21] K. S. Burrowes, E. A. Hoffman, and M. H. Tawhai, “Species-specific pulmonary arterial asymmetry determines species differences in regional pulmonary perfusion,” (in eng), *Ann Biomed Eng*, vol. 37, no. 12, pp. 2497 – 509, Dec 2009, 10.1007/s10439-009-9802-2 10.1007/s10439-009-9802-2 PMC3056879 19768544

[22] Y. C. Lee et al., “MDCT-based quantification of porcine pulmonary arterial morphometry and self-similarity of arterial branching geometry,” (in eng), *J Appl Physiol (1985)*, vol. 114, no. 9, pp. 1191–201, May 2013, 10.1152/japplphysiol.00868.2012 23449941 10.1152/japplphysiol.00868.2012 PMC4074002

[23] L. Goubergrits et al., “CT-based comparison of porcine, ovine, and human pulmonary arterial morphometry,” (in eng), *Sci Rep*, vol. 13, no. 1, p. 20211, Nov 18 2023, 10.1038/s41598-023-47532-8 10.1038/s41598-023-47532-8 PMC10657407 37980386

[24] S. Hyun, C. Kleinstreuer, and J. P. Archie, Jr., “Hemodynamics analyses of arterial expansions with implications to thrombosis and restenosis,” (in eng), *Med Eng Phys*, vol. 22, no. 1, pp. 13–27, Jan 2000, 10.1016/s1350-4533(00)00006-0 10.1016/s1350-4533(00)00006-0 10817945

[25] V. T. Turitto and C. L. Hall, “Mechanical factors affecting hemostasis and thrombosis,” (in eng), *Thromb Res*, vol. 92, no. 6 Suppl 2, pp. S25-31, Dec 15 1998, 10.1016/s0049-3848(98)00157-1 10.1016/s0049-3848(98)00157-1 9886907

[26] S. Morel et al., “Effects of Low and High Aneurysmal Wall Shear Stress on Endothelial Cell Behavior: Differences and Similarities,” (in eng), *Front Physiol*, vol. 12, p. 727338, 2021, 10.3389/fphys.2021.727338 10.3389/fphys.2021.727338 PMC8551710 34721060

[27] A. M. Malek, S. L. Alper, and S. Izumo, “Hemodynamic shear stress and its role in atherosclerosis,” (in eng), *Jama*, vol. 282, no. 21, pp. 2035-42, Dec 1 1999, 10.1001/jama.282.21.2035 10.1001/jama.282.21.2035 10591386

[28] FDA. “Modeling & Simulation at FDA.” https://www.fda.gov/science-research/about-science-research-fda/modeling-simulation-fda (accessed 04.07, 2025).

[29] A. Asadbeygi, S. Lee, J. Kovalchin, and H. Hatoum, “Effect of Beta Blockers on the Hemodynamics and Thrombotic Risk of Coronary Artery Aneurysms in Kawasaki Disease,” (in eng), *J Cardiovasc Transl Res*, vol. 16, no. 4, pp. 852–861, Aug 2023, 10.1007/s12265-023-10370-0 36932263 10.1007/s12265-023-10370-0

[30] D. Belkacemi, M. Tahar Abbes, M. Al-Rawi, A. M. Al-Jumaily, S. Bachene, and B. Laribi, “Intraluminal Thrombus Characteristics in AAA Patients: Non-Invasive Diagnosis Using CFD,” (in eng), *Bioengineering (Basel)*, vol. 10, no. 5, Apr 27 2023, 10.3390/bioengineering10050540 10.3390/bioengineering10050540 PMC10215439 37237609

[31] S. C. Corbett, A. Ajdari, A. U. Coskun, and H. Nayeb-Hashemi, “Effect of pulsatile blood flow on thrombosis potential with a step wall transition,” (in eng), *Asaio j*, vol. 56, no. 4, pp. 290-5, Jul-Aug 2010, 10.1097/MAT.0b013e3181db2476 10.1097/MAT.0b013e3181db2476 20508499

[32] C. Trenti, M. Ziegler, N. Bjarnegård, T. Ebbers, M. Lindenberger, and P. Dyverfeldt, “Wall shear stress and relative residence time as potential risk factors for abdominal aortic aneurysms in males: a 4D flow cardiovascular magnetic resonance case-control study,” (in eng), *J Cardiovasc Magn Reson*, vol. 24, no. 1, p. 18, Mar 18 2022, 10.1186/s12968-022-00848-2 35303893 10.1186/s12968-022-00848-2 PMC8932193

[33] J. M. Dolan, J. Kolega, and H. Meng, “High wall shear stress and spatial gradients in vascular pathology: a review,” (in eng), *Ann Biomed Eng*, vol. 41, no. 7, pp. 1411-27, Jul 2013, 10.1007/s10439-012-0695-0 10.1007/s10439-012-0695-0 PMC3638073 23229281

[34] A. Balasubramanya et al., “Hemodynamics and wall shear metrics in a pulmonary autograft: Comparing a fluid-structure interaction and computational fluid dynamics approach,” (in eng), *Comput Biol Med*, vol. 176, p. 108604, Jun 2024, 10.1016/j.compbiomed.2024.108604 10.1016/j.compbiomed.2024.108604 38761502

[35] F. Kong, V. Kheyfets, E. Finol, and X. C. Cai, “Simulation of unsteady blood flows in a patient-specific compliant pulmonary artery with a highly parallel monolithically coupled fluid-structure interaction algorithm,” (in eng), *Int J Numer Method Biomed Eng*, vol. 35, no. 7, p. e3208, Jul 2019, 10.1002/cnm.3208 30989794 10.1002/cnm.3208

[36] J. Liu, W. Yang, I. S. Lan, and A. L. Marsden, “Fluid-structure interaction modeling of blood flow in the pulmonary arteries using the unified continuum and variational multiscale formulation,” (in eng), *Mech Res Commun*, vol. 107, Jul 2020, 10.1016/j.mechrescom.2020.103556 10.1016/j.mechrescom.2020.103556 PMC7405952 32773906

[37] M. S. Cabrera, C. W. Oomens, C. V. Bouten, A. J. Bogers, S. P. Hoerstrup, and F. P. Baaijens, “Mechanical analysis of ovine and pediatric pulmonary artery for heart valve stent design,” (in eng), *J Biomech*, vol. 46, no. 12, pp. 2075–81, Aug 9 2013, 10.1016/j.jbiomech.2013.04.020 23849135 10.1016/j.jbiomech.2013.04.020

[38] P. B. Matthews et al., “Comparison of porcine pulmonary and aortic root material properties,” (in eng), *Ann Thorac Surg*, vol. 89, no. 6, pp. 1981-8, Jun 2010, 10.1016/j.athoracsur.2010.03.002 10.1016/j.athoracsur.2010.03.002 20494060

[39] B. A. Zambrano et al., “Image-based computational assessment of vascular wall mechanics and hemodynamics in pulmonary arterial hypertension patients,” (in eng), *J Biomech*, vol. 68, pp. 84–92, Feb 8 2018, 10.1016/j.jbiomech.2017.12.022 10.1016/j.jbiomech.2017.12.022 PMC5783768 29310945

[40] H. Spriestersbach et al., “First percutaneous implantation of a completely tissue-engineered self-expanding pulmonary heart valve prosthesis using a newly developed delivery system: a feasibility study in sheep,” (in eng), *Cardiovasc Interv Ther*, vol. 32, no. 1, pp. 36–47, Jan 2017, 10.1007/s12928-016-0396-y 10.1007/s12928-016-0396-y 27139179

[41] M. Lipiski et al., “Computed Tomography-based evaluation of porcine cardiac dimensions to assist in pre-study planning and optimized model selection for pre-clinical research,” (in eng), *Sci Rep*, vol. 10, no. 1, p. 6020, Apr 7 2020, 10.1038/s41598-020-63044-1 10.1038/s41598-020-63044-1 PMC7138799 32265478

[42] J. Brüning et al., “In-silico enhanced animal study of pulmonary artery pressure sensors: assessing hemodynamics using computational fluid dynamics,” (in eng), *Front Cardiovasc Med*, vol. 10, p. 1193209, 2023, 10.3389/fcvm.2023.1193209 37745132 10.3389/fcvm.2023.1193209 PMC10517052

[43] D. R. K. Studien. Studie zur Analyse der Parameter der internen und externen Herzleistung, des Herzzeitvolumens und aortalen Compliance mittels kardialer Magnetresonanztomographie bei Patienten mit Herzinsuffzienz [Online] Available: https://drks.de/search/de/trial/DRKS00015615

[44] A. Faragli et al., “Non-invasive CMR-Based Quantification of Myocardial Power and Efficiency Under Stress and Ischemic Conditions in Landrace Pigs,” (in eng), *Front Cardiovasc Med*, vol. 8, p. 689255, 2021, 10.3389/fcvm.2021.689255 34381823 10.3389/fcvm.2021.689255 PMC8352437

[45] A. H. Mahnken, D. Henzler, E. Klotz, A. Hennemuth, J. E. Wildberger, and R. W. Günther, “Determination of cardiac output with multislice spiral computed tomography: a validation study,” (in eng), *Invest Radiol*, vol. 39, no. 8, pp. 451-4, Aug 2004, 10.1097/01.rli.0000128655.58691.14 10.1097/01.rli.0000128655.58691.14 15257205

[46] G. J. van Essen et al., “Cardiovascular Function of Modern Pigs Does not Comply with Allometric Scaling Laws,” (in eng), *Sci Rep*, vol. 8, no. 1, p. 792, Jan 15 2018, 10.1038/s41598-017-18775-z 10.1038/s41598-017-18775-z PMC5768797 29335617

